# Metabolite-cycled density-weighted concentric rings k-space trajectory (DW-CRT) enables high-resolution 1 H magnetic resonance spectroscopic imaging at 3-Tesla

**DOI:** 10.1038/s41598-018-26096-y

**Published:** 2018-05-17

**Authors:** Adam Steel, Mark Chiew, Peter Jezzard, Natalie L. Voets, Puneet Plaha, Michael Albert Thomas, Charlotte J. Stagg, Uzay E. Emir

**Affiliations:** 1 Wellcome Centre for Integrative Neuroimaging, FMRIB Division, Nuffield Department of Clinical Neurosciences, University of Oxford, Oxford, OX3 9DU UK; 2 Laboratory of Brain and Cognition, National Institute of Mental Health, National Institutes of Health, Bethesda, MD 20814 USA; 3 Department of Neurosurgery, John Radcliffe Hospital, Oxford, United Kingdom; 4 Department of Radiological Sciences, University of California, Los Angeles, California, USA; 5 Oxford Centre for Human Brain Activity, Wellcome Centre for Integrative Neuroimaging, Department of Psychiatry, University of Oxford, Oxford, OX3 7JX UK; 6 Purdue University, School of Health Sciences, West Lafayette, IN 47907 USA

## Abstract

Magnetic resonance spectroscopic imaging (MRSI) is a promising technique in both experimental and clinical settings. However, to date, MRSI has been hampered by prohibitively long acquisition times and artifacts caused by subject motion and hardware-related frequency drift. In the present study, we demonstrate that density weighted concentric ring trajectory (DW-CRT) k-space sampling in combination with semi-LASER excitation and metabolite-cycling enables high-resolution MRSI data to be rapidly acquired at 3 Tesla. Single-slice full-intensity MRSI data (short echo time (TE) semi-LASER TE = 32 ms) were acquired from 6 healthy volunteers with an in-plane resolution of 5 × 5 mm in 13 min 30 sec using this approach. Using LCModel analysis, we found that the acquired spectra allowed for the mapping of total N-acetylaspartate (median Cramer-Rao Lower Bound [CRLB] = 3%), glutamate+glutamine (8%), and glutathione (13%). In addition, we demonstrate potential clinical utility of this technique by optimizing the TE to detect 2-hydroxyglutarate (long TE semi-LASER, TE = 110 ms), to produce relevant high-resolution metabolite maps of grade III IDH-mutant oligodendroglioma in a single patient. This study demonstrates the potential utility of MRSI in the clinical setting at 3 Tesla.

## Introduction

Magnetic resonance spectroscopy (MRS) is a quantitative MR technique that allows neurochemicals to be accurately assayed *in vivo* in humans^1^. A wide variety of neurochemicals can be detected by MRS at 3 Tesla, including N-acetylaspartate (NAA), creatine (Cr), choline (Cho), *myo*-inositol (*myo*-Ins), and glutamate/glutamine (Glu/Gln). MRS can be used to characterize the progression of neurodegenerative disease^1^, for example to conduct non-invasive biopsies to distinguish between types of tumors^2,3^. An extension of single-voxel (SV) MRS is magnetic resonance spectroscopic imaging (MRSI), which allows neurochemical profiles to be acquired from multiple voxels simultaneously over substantial regions of the brain^1^. By acquiring multiple voxels simultaneously, MRSI has obvious advantages over SV spectroscopy for both research and clinical purposes, but this technique has been hampered by several factors, including relatively long acquisition times^4^, side lobe artifacts^5^, eddy-current-induced artefacts, and tracking ***B***_0_ drifts due to subject motion or thermal fluctuation. Addressing these issues could result in an increased use of this technique in both clinical and research settings.

Several advances in MRSI acquisition and preprocessing have been made that address each of these issues independently. For example, sampling k-space using a density-weighted (DW) pattern to shape the spatial response function (SRF)^5–7^ can help to improve side lobe artefacts and improve the spatial autocorrelation of the acquired image. Although DW methods implemented with conventional phase encoding may result in long acquisition times, fast acquisition trajectories such as echo-planar spectroscopic imaging (epsi)^8–10^ or spiral^7^ offer a significant reduction in acquisition time and SRF side lobe contamination^11^. For fast MRSI, concentric ring trajectory (CRT) has been demonstrated to be superior, as this trajectory is less susceptible to gradient timing distortions compared to spiral trajectories, and is more time efficient than other EPI-based trajectories^12–14^. Although we, and others, have previously demonstrated the feasibility of DW-CRT^5,15^ at ultra-high field (≥7 T), this has yet to be shown at 3 Tesla. This advancement would be important given the wide-spread availability of 3 Tesla MRI scanners compared to ultra-high field.

In addition to advances in k-space sampling schemes, one key advance that could improve the utility of MRSI is non-water suppressed metabolite-cycling^14,16–19^. Metabolite-cycling incorporates both upfield and downfield signal acquisitions, which enables the acquisition of a full-SNR water image and metabolite signals simultaneously. The simultaneous acquisition of water and metabolite images, enables correction of gradient-induced sideband modulations, eddy-current-induced artefacts and tracking ***B***_0_ drifts due to subject motion or scanner drift. In single voxel MRS, this technique dramatically improves SNR even in the case of severe motion^16^.

The present study therefore sought to integrate these techniques and demonstrate the feasibility of metabolite-cycled^14^ full-intensity short-TE semi-LASER MRSI using the DW-CRT acquisition. In six healthy volunteers, we show that, when used in combination with advanced lipid-removal during post-processing^20,21^, this method achieves high-resolution and high spectral quality MRSI at 3 Tesla across a large brain region that extends out to the regions near the subcutaneous lipid layer. Finally, we demonstrate the potential clinical feasibility by mapping (0.25 mL) 2-hydroxglutarate (2-HG) in a patient with a grade III brain tumor.

## Methods

### Validation study

Six healthy volunteers [three males/three females, 24.5 ± 2.07 years (mean ± std)] participated in the first part of the study. All subjects gave informed consent under an institutionally approved technical development protocol at the University of Oxford in accordance with the Declaration of Helsinki.

### MRI data acquisition

Scans were acquired using a Siemens Prisma 3-Tesla (Siemens, Erlangen, Germany) whole body MRI scanner (maximum gradient of 80 mT/m and maximum slew rate of 200 mT/m/ms) with a 32-channel (N_channels_) head array receive coil. To enable MRSI grid placement, a T_1_-weighted MP-RAGE dataset (T_R_ = 1900 ms, T_E_ = 3.97 ms, T_I_ = 904 ms, flip angle = 8°, 192 transverse slices, 1 mm^3^ isotropic voxels) was acquired for each subject. GRESHIM (gradient-echo shimming) was used for B_0_ shimming^22^.

### Density-weighted CRT k-space trajectory

The DW acquisition method relies on varying the density of sampled points in k-space^11^ to create an effective windowing of the data. A Hanning-window density weighting was used to determine the radius of each ring, since it has been shown to provide a good compromise between sensitivity and spatial resolution^23^. The function used is given by:

1 $$w(k)=\frac{{\rm{\Delta }}x}{2}(1+\,\cos (2\pi k{\rm{\Delta }}x/\alpha ))$$

where Δx is the nominal spatial resolution. In this equation, the parameter α controls the adjustment of the effective spatial resolution by modulating the width of the SRF main lobe. When α is set to 1, DW-CRT spreads between −1/(2∆x) and 1/(2∆x).

### Non-water-suppressed metabolite cycling MRSI acquisition

**Figure 1:**
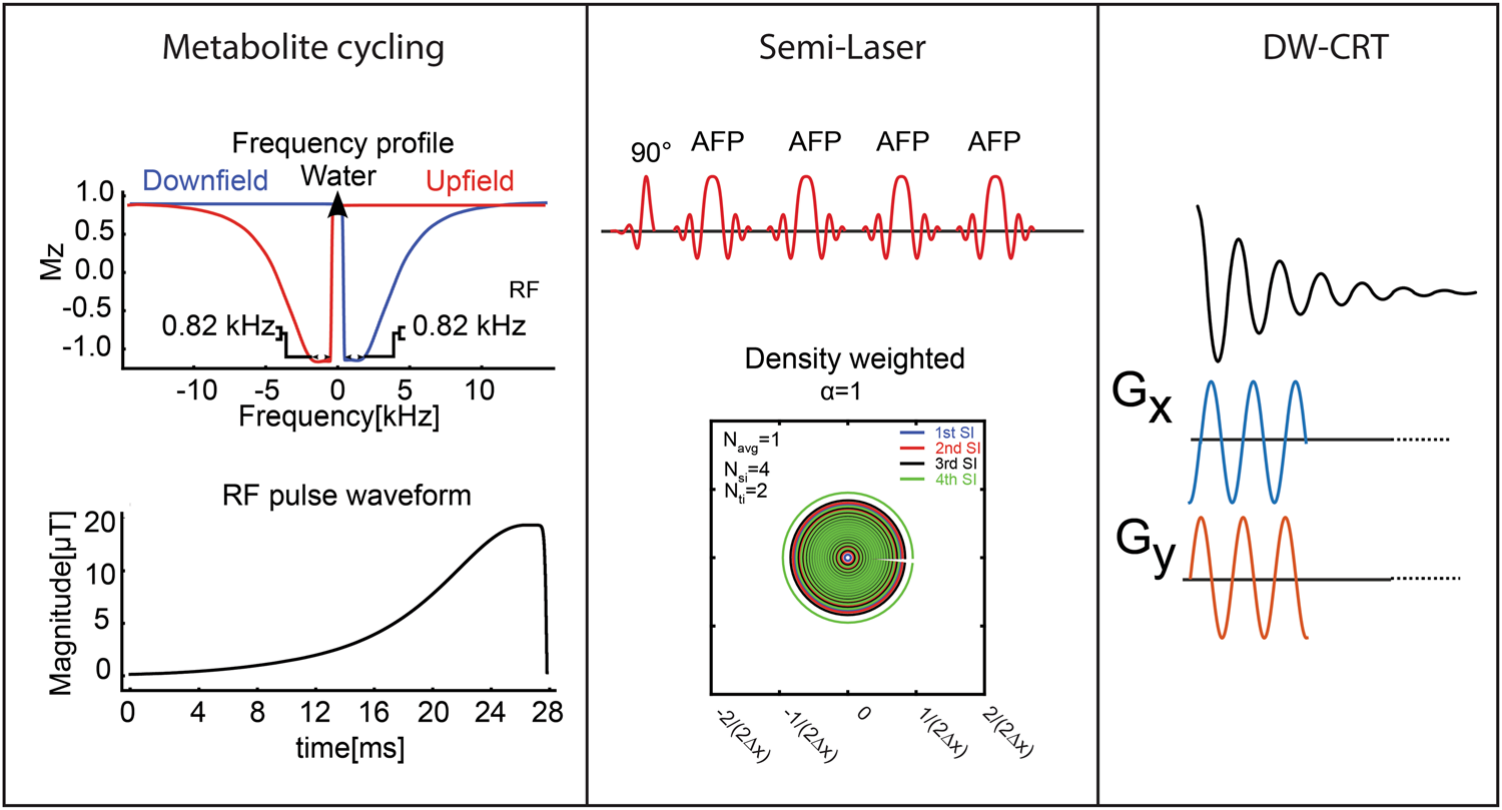
Pulse sequence diagram of the proposed density-weighted non-water suppressed semi-LASER MRSI acquisition method. Prior to semi-LASER localization, narrow-band adiabatic RF pulses invert the spectral range where metabolite signals are expected either upfield or downfield with respect to water. A density-weighted semi-circular trajectory was then used to sample k-space.

Non-water-suppressed metabolite-cycling MRSI was acquired using parameters described in Emir *et al*.^14^. Two asymmetric narrow transition-band adiabatic RF pulses with mirrored inversion profiles were applied in alternating TRs for the inversion of the upfield and downfield (relative to water) spectral resonances 9.6 ms before the semi-LASER localization^24^. An 80 Hz transition bandwidth (−0.95 < M_z_/M_0_ < 0.95) and 820 Hz inversion bandwidth (−1 < M_z_/M_0_ < −0.95), 70 to −750 Hz) downfield/upfield from the carrier frequency was achieved using a maximum B_1_ of 19 µT and pulse duration (T_P_) of 27 ms. The center of the transition band (M_z_ = 0) was placed at the carrier frequency offset by +60 Hz and −60 Hz for downfield and upfield, respectively. The pulse sequence was delivered with adiabatic pulse parameters: hyperbolic secant pulse, HS_1/2_, with R = 10 and 0.9 × T_p_, tanh/tan pulse with R = 40 and 0.1 × T_P_)^14^. The description of the pulse sequence can be found in Fig. 1.

Localization and acquisition of MRSI data used methods developed at 7 T^5^. 2D MRSI scans were collected from a single slice manually positioned using the T_1_-weighted MP-RAGE image. The imaging box was positioned to include motor cortex, frontal cortex, cingulate cortex, and parietal cortex, while avoiding the lateral ventricles. A 2D concentric *k*-space trajectory was used to sample polar *k*-space data^12^. To satisfy the requirement of avoiding azimuthal aliasing^25^, 64 points per ring were collected with an ADC bandwidth of 80 kHz and a maximum slew rate = 138.5 mT/m/ms. 512 spectral points (N_sp_) were collected with an effective spectral bandwidth of 1250 Hz. Thus, a high in-plane resolution (5 mm × 5 mm) with thickness 10 mm was achieved using FOV = 240 mm × 240 mm × 10 mm, semi-LASER localization = 145 mm × 120 mm × 10 mm, TR = 1350 ms, TE = 32 ms, 4 spatial interleaves (N_interleaves_), N_directions_ = 2 (upfield/downfield), N_ring_ = 24, (TR × N_interleaves_ × N_ring_ × N_directions_ = 259.2 s)^24,26^. The fully excited volume extended beyond the boundary of the superficial lipid. Rather than use outer-volume suppression pulses, the artifacts caused by including the superficial lipids were corrected during image processing, described below. Three independent scans were acquired and averaged together to form the final image set (total acquisition time approximately 13 min 30 s).

### Post-processing

Reconstruction algorithms were implemented in MATLAB (MathWorks, Natick, MA, USA). NUFFT gridding was performed without using any post-hoc density compensation, since this is not required for DW-CRT data. The final matrix size of the reconstructed MRSI image after NUFFT was 2N_ring_ × 2N_ring_ × N_sp_ × N_channels_ × N_directions_.

Upfield and downfield single-shot non-water-suppressed FIDs were combined as described in Emir, *et al*.^14^. Odd (upfield) and even (downfield) single-shot non-water-suppressed FIDs edited by asymmetric RF pulses were corrected on the basis of the water signal. The frequency correction was performed using a cross-correlation algorithm and phase correction was performed using a least squares fit algorithm. Upfield (S_a_) and downfield (S_b_) edited spectra were summed and used to remove residual eddy current effects^27^, combine the phased-array coil spectra^28^. Metabolite spectra were subsequently calculated by subtracting the alternating FIDs. Differences in the water peak amplitude between upfield (S_a_) and downfield (S_b_) edited spectra was corrected using the method reported in^29^. The residual water peak was filtered with the Hankel-Lanczos singular value decomposition (HLSVD) algorithm^30^.

Following HLSVD filtering, the lipid contamination was removed during post-processing using a lipid-basis penalty algorithm^20,21^. Briefly, an iterative lipid-basis reconstruction with L2-penalty with a regularization parameter of 10^−3^ was applied to the metabolite image by assuming that the metabolite spectra from brain and lipid spectra from the lipid masks were orthogonal. Non-water-suppressed single-slice MRSI data were used to generate a lipid mask, i.e. of skull + subcutaneous fat, to generate the lipid basis set, and a brain mask for this process. These masks were hand-drawn based on the observed contrast between the brain and non-brain tissue in the water and metabolite images. The voxels processed within the brain mask were then passed to LCModel for analysis.

The LCModel package was used to quantify the metabolite spectrum for each MRSI voxel^31^. The model spectra of alanine (Ala), aspartate (Asp), ascorbate/vitamin C (Asc), glycerophosphocholine (GPC), phosphocholine (PC), creatine (Cr), phosphocreatine (PCr), GABA, glucose, glutamine (Gln), glutamate (Glu), glutathione, lactate (Lac), *myo*-Inositol (*myo*-Ins), NAA, N-acetylaspartylglutamate, phosphoethanolamine (PE), scyllo-Inositol (*scyllo*-Ins) and taurine were generated based on previously reported chemical shifts and coupling constants by the GAMMA/PyGAMMA simulation library of VeSPA (Versatile Simulation, Pulses and Analysis) according to a density matrix formalism^32^. Simulations were performed using the same RF pulses and sequence timings as those on the 3 T system in use. Eight LCModel-simulated macromolecule resonances were included in the analysis at the following positions: 0.91, 1.21, 1.43, 1.67, 1.95, 2.08, 2.25 and 3 ppm. If the correlation between two metabolites was consistently high (correlation coefficient < −0.5) in a given region, their sums are reported, e.g. combined glutamate and glutamine (Glu + Gln, Glx), total creatine (Cr + PCr, tCr) and total choline (GPC + PC, tCho).

### Gray matter probability assessment

In the interest of validating the sensitivity of density weighted single-slice MRSI at 3 T, we calculated the Glx:tCr and tCho:tCr ratios relative to voxel-wise gray matter probability estimates for each subject. Glx:tCr and tCho:tCr were chosen based on their strong association with gray matter and white matter, respectively^33,34^. Therefore, we hypothesized the opposite pattern of relationship between gray-matter concentration for these metabolites, given the evidence that white matter has elevated tCho:tCr in comparison with gray matter whereas Glx:tCr is higher in the region of gray matter^9,35–37^. To calculate gray matter probability, the T1-weighted image for each subject was segmented into gray and white matter tissue compartments using the segment function in SPM 8^38^. The segmented image was resampled to the MRSI resolution using linear interpolation. The gray matter probability of each voxel was correlated to the tCho:tCr and Glx:tCr ratios using Pearson’s correlation. Voxels selected for this analysis were restricted to those with CRLB values below 50 for the respective metabolite maps.

### Clinical feasibility study

A 72-year-old, right handed female presented with a three-month history of tinnitus in January 2017. Clinical diagnostic MRI revealed an intra-axial mass involving most of the left temporal lobe, containing an area of enhancement consistent with a grade III glioma. A subsequent biopsy confirmed an IDH-mutant oligodendroglioma. For technical details of the biopsy see^39^. The patient was offered an awake resection and gave fully informed written consent to take part in research imaging investigations at the University of Oxford FMRIB Centre prior to resection. The study was approved by the South Central Oxford B Research Ethics Committee.

A high-resolution structural T1-weighted sequence was acquired to delineate the tumor and inform MRSI voxel placement. MRSI data were acquired with parameters consistent with the healthy volunteer acquisition; apart from a TE of 110 ms to be sensitive to 2-HG^39^ [resolution = 5 × 5 × 10 mm^3^, N_p_ring_ = 64, FOV = 240 mm × 240 mm × 10 mm, semi-LASER localization = 145 mm × 120 mm × 10 mm, TR = 1350 ms, 4 spatial interleaves (N_interleaves_), N_directions_ = 2 (upfield/downfield), N_ring_ = 24, TR × N_interleaves_ × N_ring_ × N_directions_ = 259.2 s]. To compare the of 2-hydroxyglutarate (2-HG):tCr, total N-acetyl-aspartate (tNAA):tCr, and tCho:tCr ratios in affected and unaffected tissue, a tumor mask was constructed based on the T1-image. This tumor mask was then reflected about the midline, such that the equivalent voxels were sampled on the unaffected hemisphere.

## Results

### *In vivo* DW-CRT images

**Figure 2:**
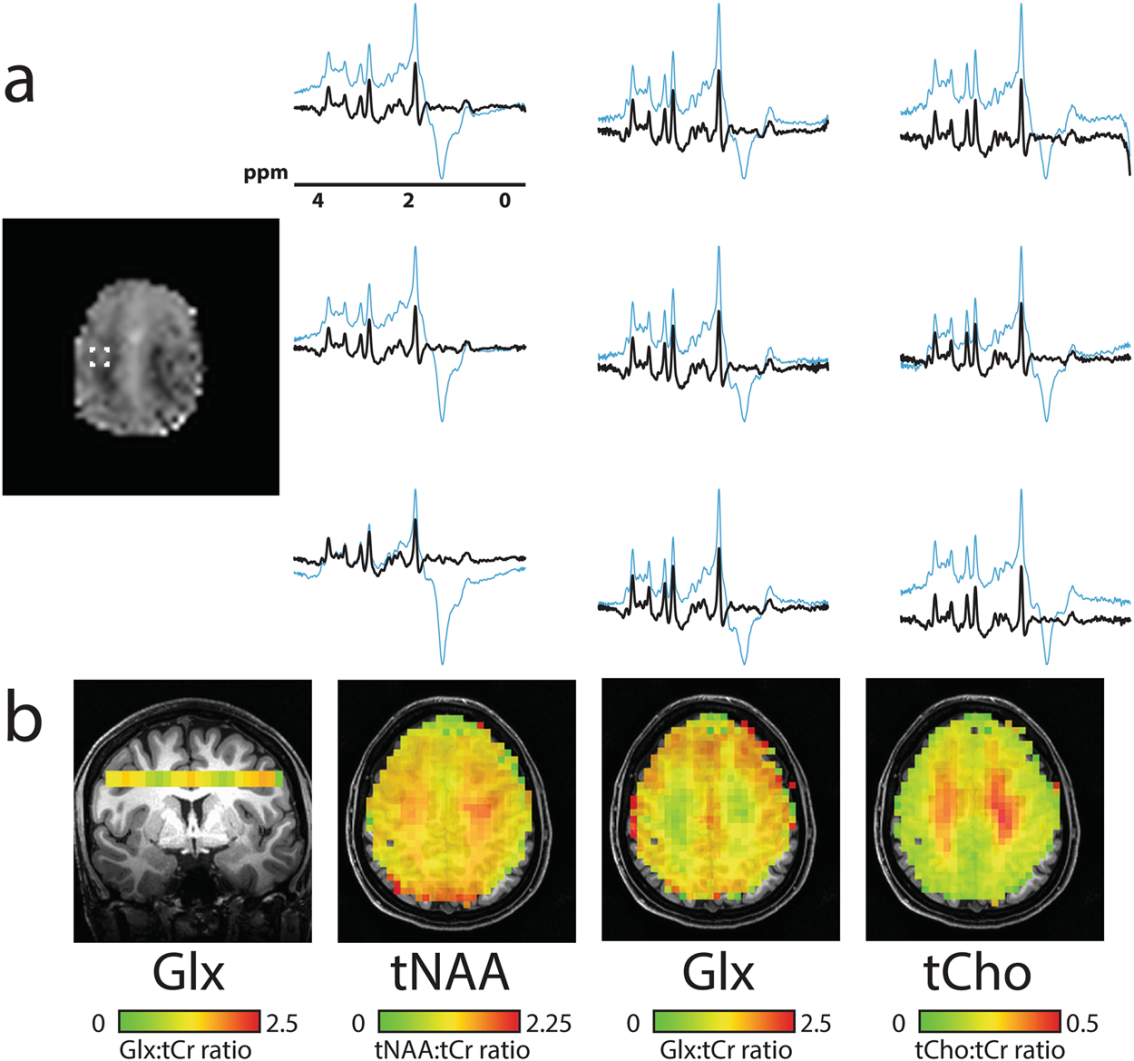
(**a**) Example spectra from nine voxels with (black) and without (blue) L2-based lipid suppression inside the region of interest marked on the single-slice image (5 × 5 × 10 mm). (**b**) Metabolite ratio distribution maps of NAA:tCr, Glx:tCr, and tCho:tCr are shown. One coronal slice (Glx:tCr) is shown to demonstrate the through plane resolution as well as the voxel level specificity of white- and gray- matter distribution. Images are shown in neurological orientation (left hemisphere on left side of the image).

**Figure 3:**
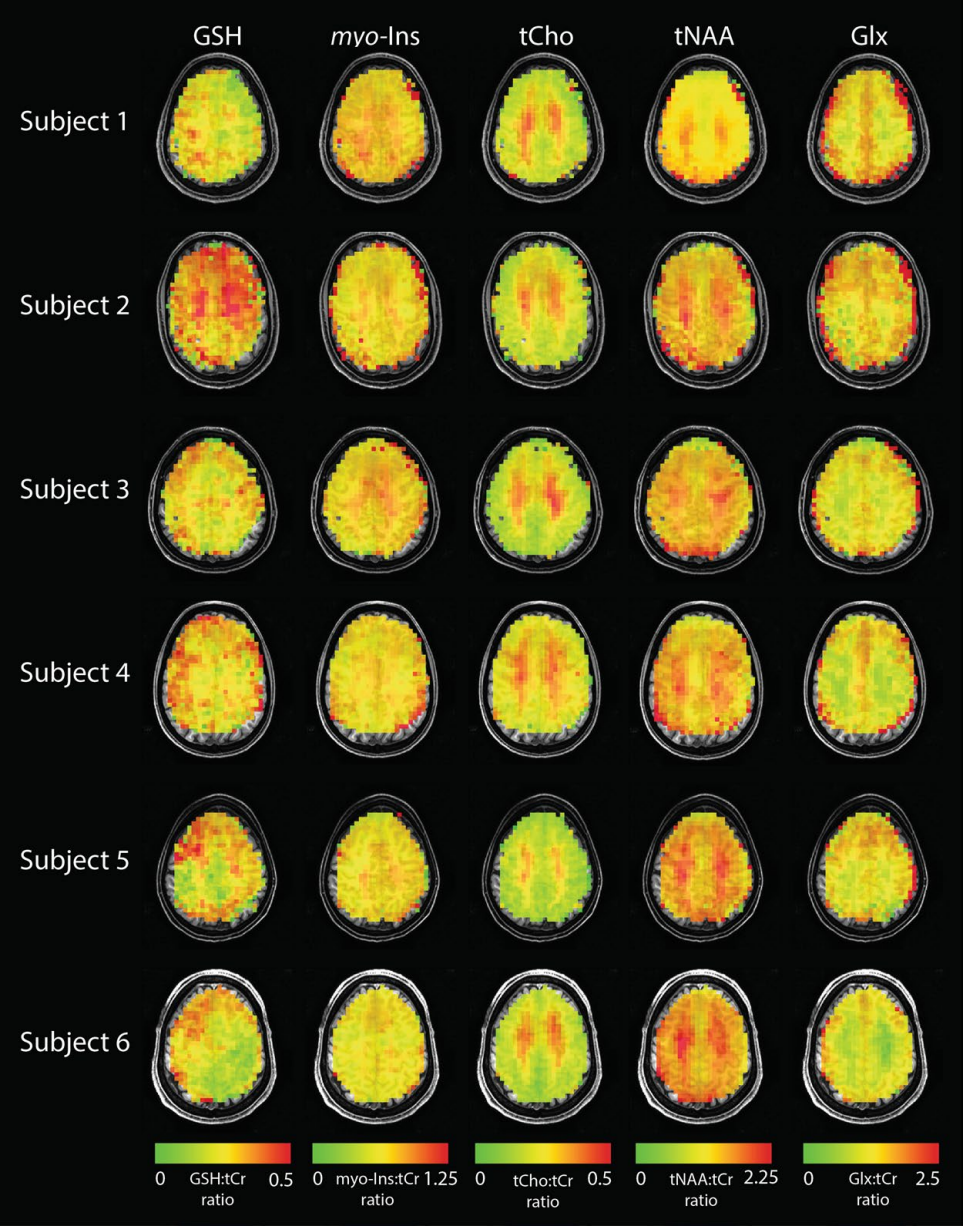
High-resolution metabolite distribution maps obtained using DW-CRT from all subjects. Ratios of GSH:tCr, *myo*-Ins:tCr, tCho:tCr, tNAA:tCr, and Glx:tCr are shown. Images are shown in neurological orientation. Non-brain tissue was masked based on the water image.

**Figure 4:**
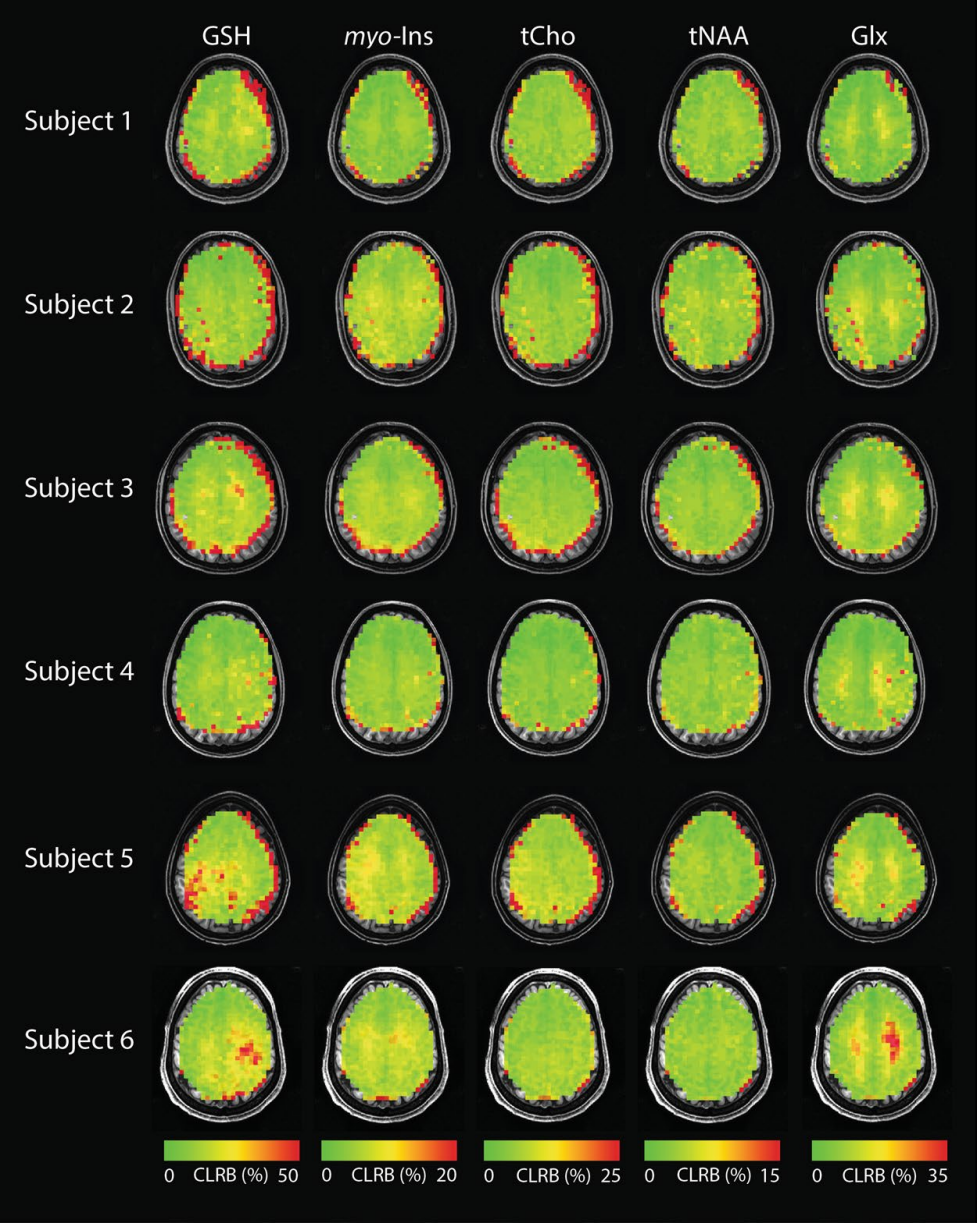
CRLB maps for GSH, *myo*-Ins, tCho, tNAA, Glx are overlaid on each subject’s anatomical image. Images are shown in neurological orientation (left hemisphere on left side of image). Non-brain tissue was masked based on the water image.

**Figure 5:**
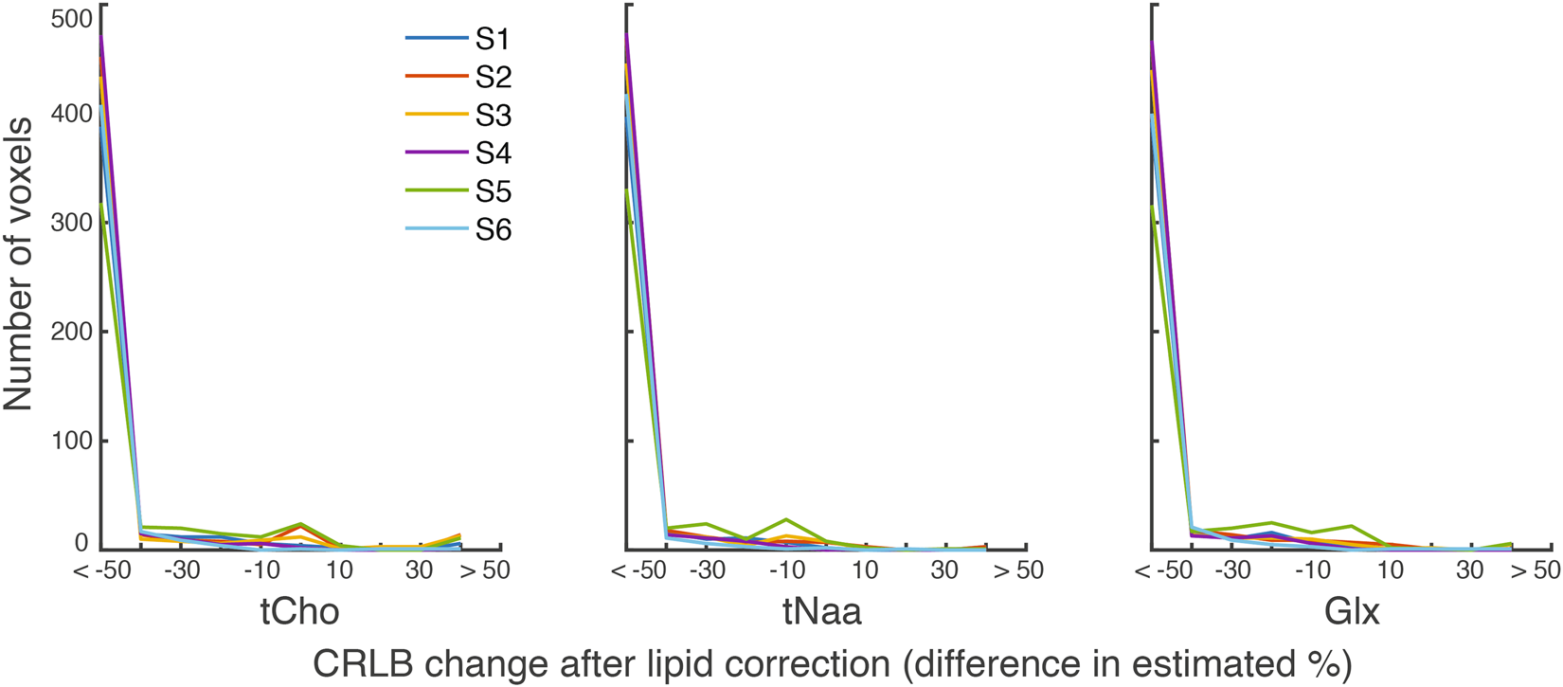
Change in LCModel fitting after lipid correction. To test the efficacy of lipid correction for improving LCModel fits, the CRLB value at each voxel was calculated for tCho, tNaa, and Glx before and after lipid correction. The difference in CRLB value was calculated. After lipid correction, the majority of voxels showed greater than 50% improvement in CRLB values. Negative values indicate an improvement in CRLB after lipid correction.

Figure 2 shows the DW-CRT spectra from a representative healthy volunteer, acquired in 13 minutes 30 seconds. As illustrated in the 3 × 3 grid, even at a high resolution (5 mm × 5 mm × 10 mm), the metabolite spectra from DW-CRT were of high quality after lipid correction. The Cramer-Rao Lower Bound (CRLB) values of six important metabolites (i.e. NAA, Cr, tCho, GSH, *myo*-Ins, and Glx) were lower than 20% in most of voxels. For each healthy control, the resulting metabolite ratio maps for voxels with CRLBs less than 20% are shown in Figs 3 and 4 shows the corresponding CRLB map. Notably, the neurochemical GABA was not reliably detected. Lipid correction substantially improved the quality of the LCModel fitting. In all subjects, lipid correction improved the CLRB values estimated by LCModel for tCho, tNaa, and Glx by greater than 50% in the majority of voxels (Fig. 5). This improvement is due to the removal of the large lipid artifacts, which would preclude spectral fitting by LCModel under normal circumstances.

**Figure 6:**
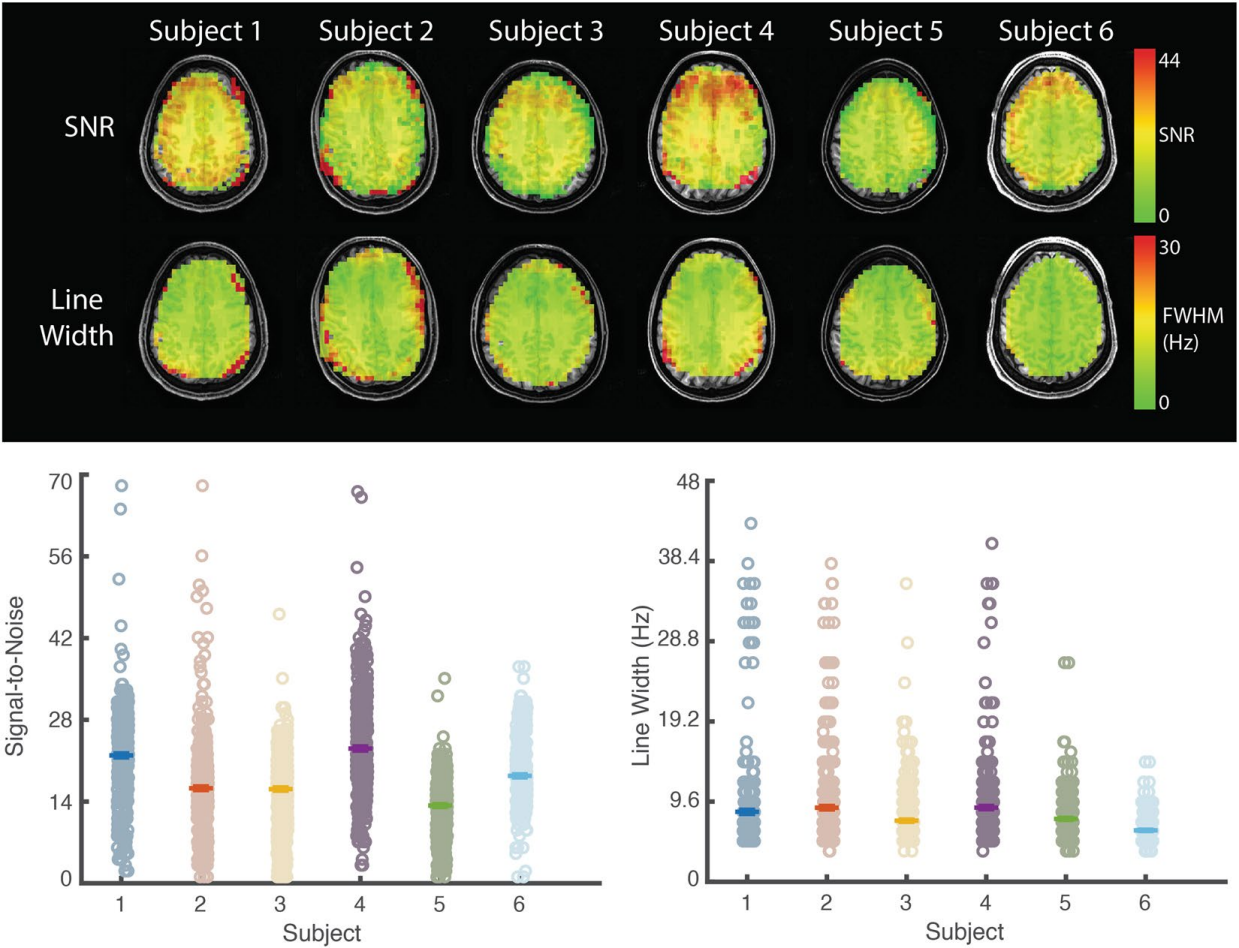
Spatial distribution of signal to noise ratio and line width across the spectroscopic image calculated by LCModel. Upper. In all subjects, both signal to noise and spectral linewidth were uniformly distributed across the image. Lower. Voxel-wise signal to noise (left) and linewidth (right) are shown. Hashes indicate subject mean. Line-width is presented in Hz. Non-brain tissue was masked based on the water image.

After lipid correction, the voxel-wise SNR and spectral linewidth calculated by LCModel did not show a systematic spatial bias (Fig. 6, upper). Across all subjects, the median voxel-wise SNR was 18 (interquartile range = 14–22) and linewidth was 7.26 Hz (interquartile range = 6.16–9.73 Hz; Fig. 6, lower).

**Figure 7:**
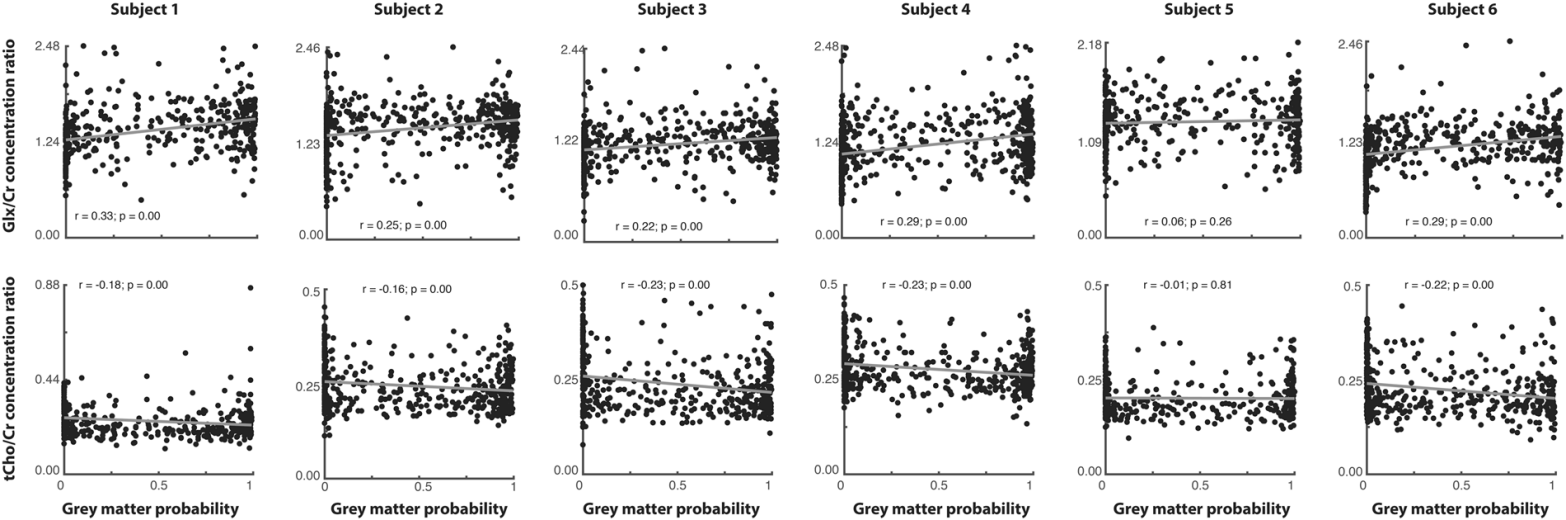
Voxel-level Glx- and tCho- to tCr ratio are correlated with voxel-wise gray-matter probability. Glx:tCr was positively associated with gray-matter probability, while tCho:tCr was negatively associated with gray-matter probability.

Prior research has suggested that the Glx^40^ and tCho^41^ should have differential distributions in gray and white matter, with Glx being higher in gray matter and tCho higher in white matter. To validate our approach, we therefore tested whether we could replicate these expected results by correlating the voxel-wise measure of gray matter probability in each spectroscopy voxel with the Glx:tCr and tCho:tCr ratios. All six subjects showed the expected positive relationship (Bonferroni-Holm corrected) between gray matter probability and Glx:tCr (Fig. 7, upper). Five out of six subjects showed the expected negative relationship between gray matter concentration and tCho:tCr (Fig. 7, lower). The subject who did not show the expected relationship between gray matter probability and tCho:tCr had the greatest mean CLRB value for tCho (median = 8%, interquartile range = 7–9%).

### Clinical feasibility

**Figure 8:**
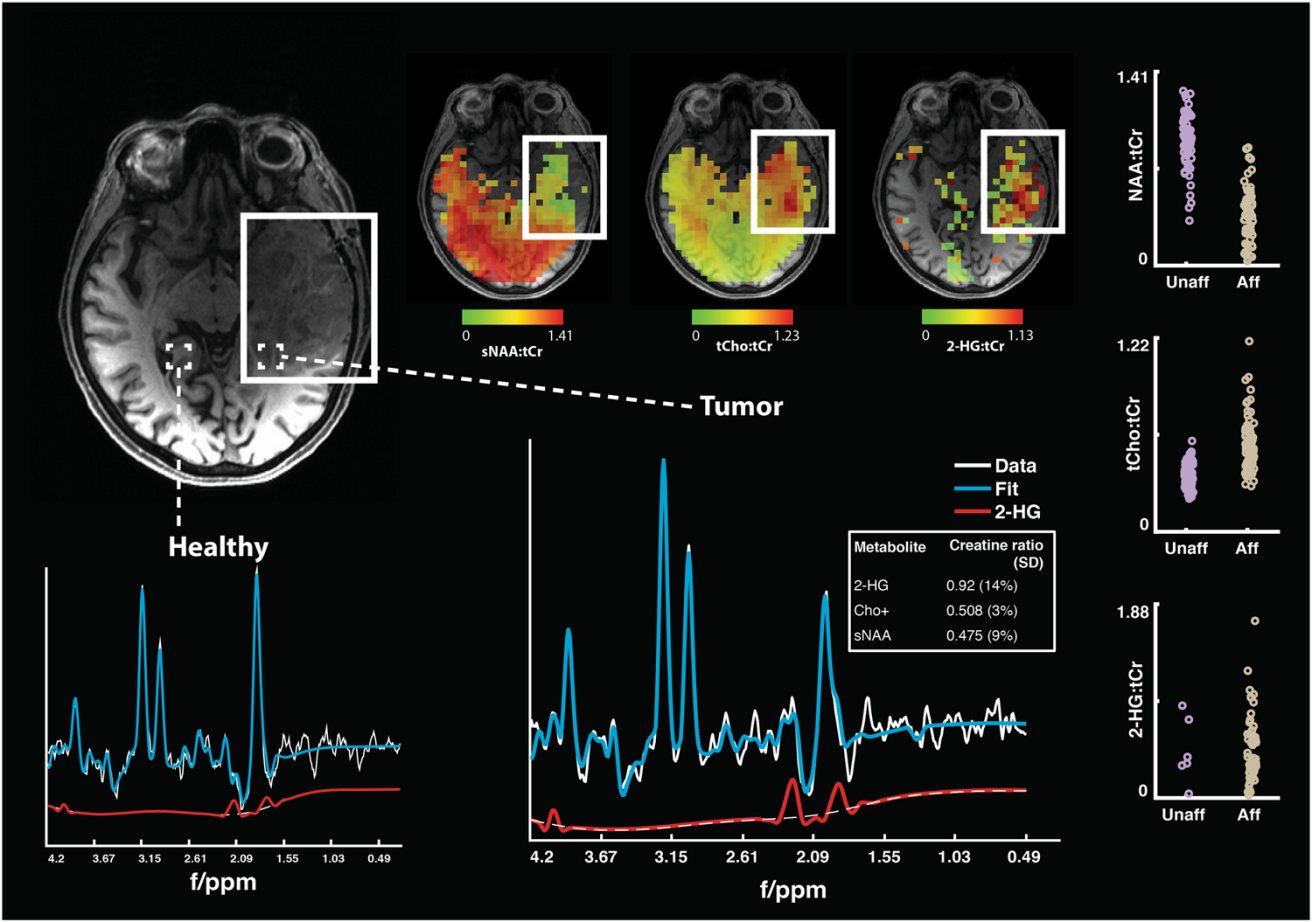
Pre-operative MRSI data acquired from a 72-year-old female with a biopsy confirmed IDH-2 mutant oligodendroglioma affecting the left temporal lobe. To assess 2-HG concentration, the TE was changed from 32 to 110 ms. All other acquisition parameters were consistent with those reported above. 2-HG:tCr, tCho:tCr, and NAA:tCr ratio maps show marked local disturbance in normal ratios in the affected area. In metabolite images, non-brain tissue was masked based on the water image.

MRSI data were acquired in a 72-year-old right handed female with a biopsy-confirmed oligodendroglioma affecting the left temporal lobe. Spectra were collected from a single-slice positioned over the center of mass of the tumor defined on a 3D T1-weighted high-resolution anatomical image (Fig. 8; acquisition time = 13 min 30 sec [3 × 4 min 30 sec]). The metabolite maps of 2-HG, tCho, and tNAA as well as a representative spectrum from healthy and affected tissue are shown in Fig. 8. As would be expected, a clear decrease in tNAA and an increase in tCho can be seen within the tumor compared with the unaffected hemisphere. In addition, 2-HG, a specific marker for IDH-mutant tumors^2,3,39^, can be seen at high concentration within the tumor but, as would be expected, is virtually absent on the contralateral side.

## Discussion

The goal of the present study was to demonstrate the feasibility and clinical potential of high-resolution fast MRSI without water suppression. We acquired data using DW-CRT k-space sampling and semi-LASER localization, which affords a substantial boost in spatial resolution while maintaining a comparable SNR and acquisition time to our previously published acquisition scheme^14^. We collected high-resolution single-slice MRSI in six healthy volunteers using a voxel size of 5 × 5 × 10 mm^3^ in 13 min 30 sec. This acquisition, in combination with advanced preprocessing techniques, yielded high-quality spectra and enabled the quantification of the important brain metabolites tNAA, tCho, GSH, *myo*-Ins, tCr, and Glx. We further demonstrated that this acquisition could be tailored to be sensitive to 2-HG, and therefore be used to delineate compromised tissue and potentially classify mutation-types in brain tumor patients. Together, these results clearly show the suitability of the proposed MRSI technique for clinical and experimental use.

The DW-CRT k-space trajectory offers significant benefits over other acquisition strategies. Compared to EPI-based trajectories and uniformly-weighted CRT trajectories, DW-CRT has been demonstrated to offer a substantial improvement in SNR at 7 T^5^. This SNR benefit comes at no detriment in acquisition time; DW-CRT has a lower acquisition time than standard EPI-based trajectories because of the improved k-space sampling efficiency. The present study did not directly compare this acquisition to others at 3 Tesla. However, one recent publication achieved a nominal voxel volume of 0.4 mL in 14 minutes at 7 Tesla, compared to the volume acquired in our study (0.25 mL) in 13.5 minutes at 3 Tesla^22^. Thus, we believe that our strategy offers substantial promise for clinical and experimental MRSI compared to other similar sequences at 3 T.

In addition, SNR is increased through the use of metabolite-cycling. Because the water-scan is implicitly acquired using this technique, eddy-current correction, correction for coil-combination, and correction for B_0_ frequency drifts caused by hardware artifacts and subject motion can all be applied^14^. Moreover, by implicitly acquiring the water image during the standard acquisition, it may be possible to achieve better between-scan alignment, which may be useful in an experimental setting. These benefits have been previously described at ultra-high field strengths^14,17,19^, and here we demonstrate the feasibility high-resolution MRSI at 3 Tesla using the semi-LASER sequence.

While this approach has great promise for clinical application, several aspects of the technique can be improved. For example, while the L2-normalization lipid removal method greatly improves the spectral quality, it does reduce the ability to measure low concentration metabolites such as GABA. In addition, the parameters for lipid removal chosen here may introduce negative inflections in the baseline which could affect LCModel quantification.

The use of the lipid penalty may also affect the quality of the absolute metabolite measures across the acquired region and reduce sensitivity to some metabolites. We therefore chose to express our concentration values relative to tCr. It is possible that lipid removal during post-processing caused the reduced differentiation in the Glx and tCho between gray and white-matter compared to previous studies^42^. Using outer volume suppression pulses to suppress the lipid contamination during acquisition could make lipid removal unnecessary. This acquisition strategy should be explored in future studies.

In conclusion, here we show that DW-CRT acquisition in combination with metabolite-cycled semi-LASER pulse localization allows fast, high-resolution MRSI to be acquired at 3 Tesla. Given the wide availability of 3 Tesla MRI scanners, this approach may have wide application in both clinical and research settings.
